# Development and validation of a nomogram for urothelial cancer in patients with chronic kidney disease

**DOI:** 10.1038/s41598-019-40276-4

**Published:** 2019-03-05

**Authors:** Che-Yi Chou, Kuo-Hsiung Shu, Hung-Chun Chen, Ming-Chang Wang, Chia-Chu Chang, Bang-Gee Hsu, Tzen-Wen Chen, Chien-Lung Chen, Chiu-Ching Huang

**Affiliations:** 1 Division of Nephrology and Kidney Institute, China Medical University and Hospitals, Taichung, Taiwan; 2 Division of Nephrology, Asia University Hospital, Taichung, Taiwan; 3 Department of Post-baccalaureate Veterinary Medicine, Asia University, Taichung, Taiwan; 4 Division of Nephrology, Taichung Veterans General Hospital, Taichung, Taiwan; 5 Division of Nephrology, Kaohsiung Medical University, Kaohsiung, Taiwan; 6 Division of Nephrology, Cheng Kung University Hospital, Tainan, Taiwan; 7 Division of Nephrology, Changhua Christian Hospital, Changhua, Taiwan; 8 Division of Nephrology, Buddhist Tzu Chi General Hospital, Hualien, Taiwan; 9 Division of Nephrology, Taipei Medical University, Taipei, Taiwan; 10 Division of Nephrology, Landseed Hospital, Zhongli, Taiwan; 11 Division of Nephrology and Kidney Institute, Department of Internal Medicine, China Medical University and Hospital, Taichung, Taiwan

## Abstract

Urothelial cancer (UC) is a common kidney cancer in Taiwan and patients with chronic kidney disease (CKD) are more at risk for UC than the general population. The diagnostic value of urine analysis and urine cytology is limited, especially in CKD patients. The aim of the study is to develop a nomogram to predict the risk of UC in CKD patients. We enrolled 169 UC patients and 1383 CKD patients from 9 hospitals in Taiwan between 2012 and 2015. CA125, HE4, clinical characteristics, and medical history were analyzed using multivariable logistic regression for its association with UC. A nomogram was developed to predict the risk of UC and was validated using Bootstrap. CA125 was associated with UC in CKD patients (OR: 5.91, 95% CI: 3.24–10.77) but HE4 was not (OR: 1.29, 95% CI: 0.67–2.35). A nomogram based on patients’ age, estimated glomerular filtration rate, CA125 (log transformed), smoking, exposure of environmental toxin, use of nonsteroid anti-inflammatory drugs, and use of traditional Chinese medicine was conducted. The AUC of the nomogram was 0.90 (95% CI: 0.86–0.92, p < 0.01). Serum CA125 may identify UC patients from CKD patients but has limited diagnostic value due to low sensitivity. The diagnostic value of serum CA125 level can be improved by the combination with clinical characteristics including age, renal function, and medical history.

## Introduction

Urothelial carcinoma (UC) is common cancer in chronic kidney disease (CKD) patients^1^. The incidence of UC in Taiwan is higher than that of other parts of the world with an unusually high incidence of 50 per 100,000 person-years^2–4^. The development of UC can be associated with multiple factors such as smoking^5^, drinking groundwater that contains heavy metals^6^, exposures to environmental toxins such as dye^7^ or organic solvent^8^, and Chinese herbs that contain aristolochic acid (AA). Aristolochic acid can be the cause of both CKD and UC^9–12^. Epigenomic factors such as CKD^13^ and exposure to heavy metals^14^, aristolochic acid^12,15^, other environmental carcinogen exposures^5^ are strongly associated with UC. Urinalysis and urine cytology are the major screening tools in a clinical setting; however, the diagnostic performance of these tests are poor^16,17^. Serum markers such as Carcinoma antigen (CA125)^18–22^ and human epididymis protein 4 (HE4)^23^ are potential serum markers for the diagnosis of UC. The serum CA125 and HE4 can be elevated in CKD patients because the removal of these serum proteins is decreased with the decline of renal function^24–27^. The diagnostic value of CA125 and HE4 for UC in CKD patients is unclear. We aimed to develop a nomogram using serum biomarkers (CA125 and HE4), and clinical variables (such as age, gender, estimated glomerular filtration rate (eGFR), medical history for early detection of UC in CKD patients. As the medical history such as exposure to herbs and groundwater that contains heavy metals may be specific to endemic regions, the application of the nomogram may be limited to the endemic regions.

## Methods

### Study population and patient recruitment

This ongoing prospective, multi-center study of urothelial cancer (UC) was initiated by Taiwan Urothelial Cancer Consortium (TUCC) aiming to investigate the risk factors of UC with multiple risk domains (genes and environments). CKD patients without UC were recruited as a control group. The TUCC was coordinated by the Kidney Institute of China Medical University Hospital (Taichung, Taiwan) and the study was proposed to nephrology and urology divisions of the other nine hospitals. These hospitals had a diverse health care level from tertiary settings to local hospitals, agreed to participate in this study, which started the patient recruitment since July 2013. The consortium affiliated centers distributed throughout the country; four were in Northern Taiwan, 3 in Central Taiwan, 2 in Southern Taiwan, and 1 in Eastern Taiwan.

UC patients older than 20 years were identified consecutively in the urology department of each hospital and defined as adult patients with new or recurrent UC. All UC cases were verified by surgical and pathological reports. Control subjects, CKD patients with no known history of malignancy, were consecutively selected from the nephrology center of each hospital. After receiving detailed explanations of the study, each of the UC cases and controls provided written informed consent for the questionnaire interview and collection of blood and urine samples.

### Ethics statement

The recruitment and follow-up protocols complied with the Declaration of Helsinki and were approved by the institutional review board of China Medical University Hospital (CMUH 102-REC2-043) and other nine hospitals.

### Data collection

**Figure 1:**
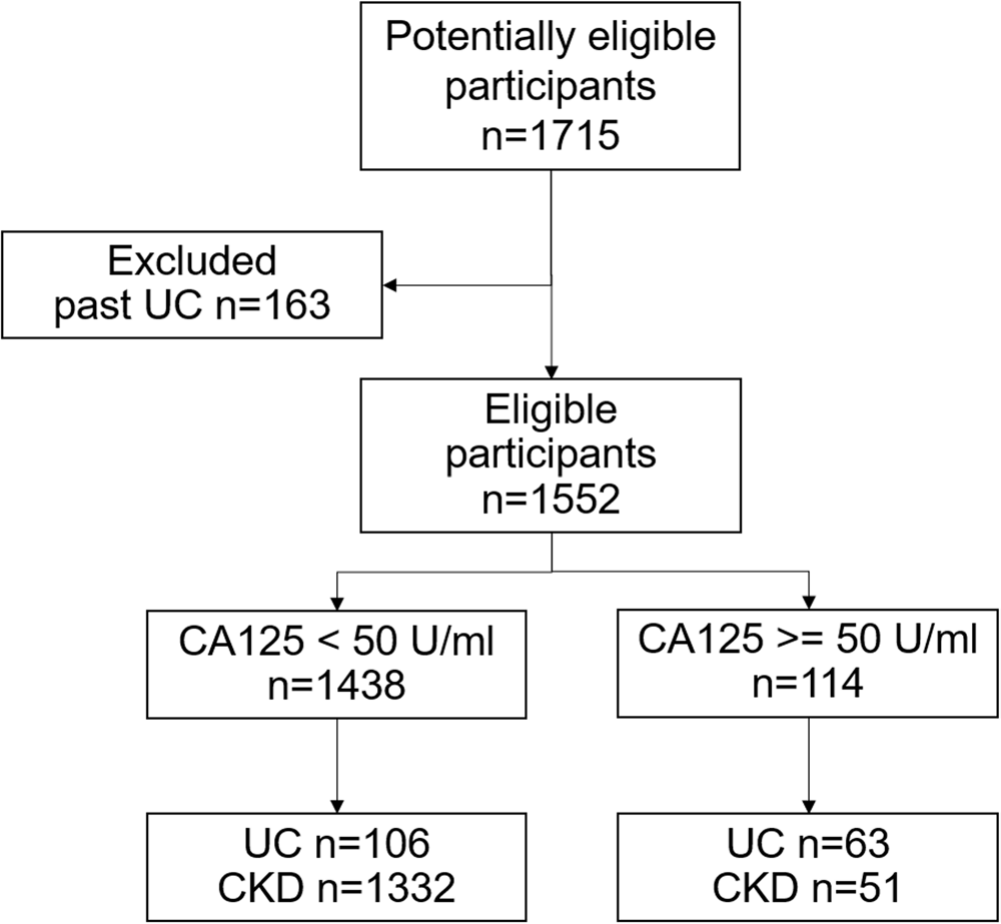
Flow chart of the study participants.

From July 2013 to December 2015, 1715 patients were enrolled and 163 patients with past UC who had no evidence of recurrence were excluded from the analysis (Fig. 1). All blood and urine were collected at enrollment. For UC patients, blood and urine samples were collected before surgical interventions.

### Biomedical measurements

CA125 and HE4 were measured at the diagnosis of UC in the UC patients and at the enrollment in the CKD patients. The measurements of CA125 and HE4 were performed in a central laboratory using an electrochemiluminescence immunoassay on Cobas e411 Elecsys 2010 (Roche Diagnostics GmbH, Germany). Body mass index (BMI), serum blood urea nitrogen (BUN), serum creatinine, estimated glomerular filtration rate (eGFR using CKD-EPI formula), serum uric acid, and serum albumin were measured.

### Environmental exposures

Smoking was defined as a history of smoking >2 pack-years and/or smoking in the last year^28^. Alcohol consumption was defined as ≥1 alcoholic drink per month^29^. Groundwater use was defined as patients who reported a history of using groundwater as a source of drinking water for more than 6 months. Exposure to dye was defined as occupational exposure to dye for more than 6 months^30^. Nonsteroid Anti-inflammatory Drugs (NSAIDs) use was defined as ingestion of NSAIDs more than four times per week^31^. Use of traditional Chinese medicine (TCM) was defined as patients who had taken Chinese herbal remedies more than three times per year.

### Statistical Analysis

Data are reported as the mean ± standard deviation, median (interquartile range, IRQ), or frequency (percentage), as appropriate. All continuous variables were tested for normality using the skewness and kurtosis test. Data were analyzed using the *t-*test for normally distributed variables, the Mann-Whitney U test for non-normalized variables, or the chi-squared test for categorical variables. The diagnostic value of CA125 and HE4 for UC was analyzed using receiver operating characteristic (ROC) analysis and the area under the ROC curve (AUC) was calculated. The cut-off of CA125 was 35 U/ml and the cutoff of HE4 was 150 pmol/L for the diagnosis of ovarian cancer. The optimal cutoff of CA125 and HE4 for the diagnosis of UC may be higher because CKD patients were enrolled as controls in this study. The optimal cutoff for the diagnosis of UC was determined based on the results of ROC analysis. Possible risk factors of UC were analyzed using univariable logistic regression, followed by multivariable logistic regression. Odds ratios (ORs) and 95% confidence intervals (CIs) of OR were calculated. The factors associated with UC in multivariable logistic regression were used to generate a nomogram for UC. All analyses were performed using Stata (StataCorp. 2013. Stata Statistical Software: Release 13. College Station, TX: StataCorp LP.). The nomogram was developed using nomolog program for Stata^32^ and the nomogram was validated using rms packages of R software with bootstrap. Values with *p* < 0.05 were considered statistically significant.

## Results

### Patient characteristics

From 2013 to 2015, 1715 patients were enrolled and 163 patients with past UC who had no evidence of recurrence were excluded from the analysis (Fig. 1). For control patients, blood and urine were collected at enrollment. For UC patients, blood and urine samples were collected before surgical interventions. One hundred and sixty-nine UC patients and 1383 CKD patients were analyzed in this study (Fig. 1 and Table 1). UC patients (mean age: 66 ± 11 years) were older than CKD patients (57 ± 13 years, p < 0.01). The BMI of UC patients was lower than that of CKD patients (p < 0.01). The CA125 (median: 18.7 U/ml, IRQ: 9.9–88.7 U/ml) of UC patients was significantly higher than that of CKD patients (median: 11.7, IRQ: 7.5–17.9, p < 0.01, Mann-Whitney U test). The HE4 was not different between UC and CKD patients. The eGFR of CKD patients was significantly lower than that of UC patients (p = 0.02). The proportion of patients with smoking (p < 0.01), use of NSAIDs (p < 0.01), with history of groundwater use (p = 0.01), exposure to toxins (p < 0.01), and use of TCM (p < 0.01) were significantly higher in UC patients than in CKD patients.

**Table 1:** Clinical Characteristics of patients.

Characteristics	UC N = 169		CKD N = 1383		P
Age (year)	67	±11	57	±13	<0.01
Male gender n%	109	64.5	800	57.8	0.85
BMI (kg/m^2^)	24.1	±3.7	24.9	±4.5	0.05
CA125 (U/ml)	18.7	9.9–88.7	11.2	7.0–17.5	<0.01
HE4 (pmol/L)	178.5	89.8–403.6	136.6	82.7–296.7	0.43
BUN (mg/dl)	27	±20	34	±25	<0.01
Creatinine (mg/dl)	2.6	±3.1	2.8	±3.1	0.40
eGFR (ml/min/1.73 m^2^)	51	±30	44	±27	<0.01
Uric acid (mg/dl)	6.6	±1.7	6.5	±1.7	0.63
Albumin (mg/dl)	3.9	±0.7	4.0	±0.6	0.92
Smoking (n/%)	34	20.1	105	7.6	<0.01
Alcohol (n/%)	20	11.8	126	9.1	0.40
NSAIDs	37	21.9	44	3.2	<0.01
Groundwater (n/%)	13	7.7	53	3.8	0.01
Toxins (n/%)	61	36.1	102	7.4	<0.01
TCM (n/%)	65	38.5	86	6.2	<0.01

BMI: body mass index, HE4: human epididymis protein 4, BUN: blood urea nitrogen, eGFR: estimated glomerular filtration rate using CKD-EPI formula, NSAIDs: Nonsteroid Anti-inflammatory Drugs, Toxins: exposure to dye, paint, or organic solvent, TCM: traditional Chinese medicine.

### Development and validation of UC nomogram

**Figure 2:**
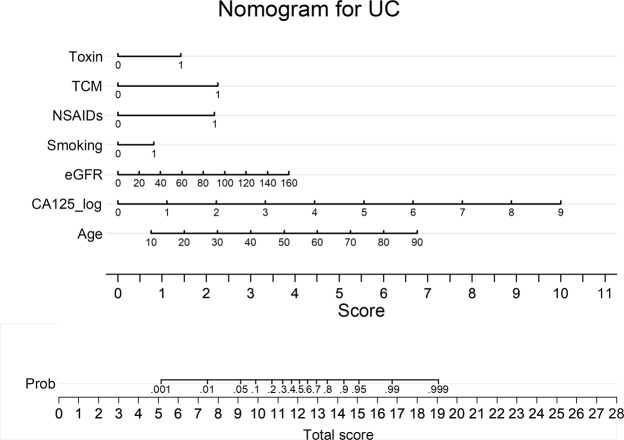
Nomogram for the diagnosis of urothelial cancer (UC). Toxin: exposure of dye, organic solvent, and paint, TCM: traditional Chinese medicine, NSAIDs: Nonsteroid Anti-inflammatory Drugs, eGFR: estimated glomerular filtration rate using CKD-EPI formula, CA125_log: log-transformed CA125, Prob: probability.

**Figure 3:**
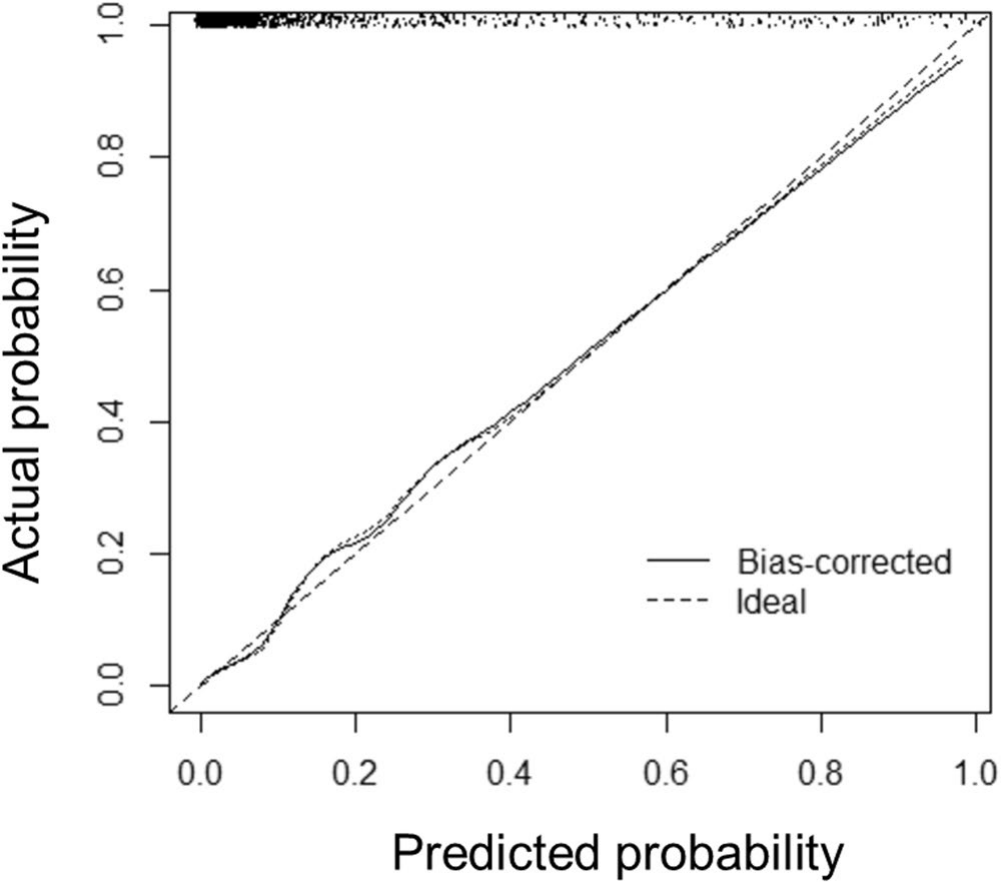
Calibration plots for the probability of urothelial cancer (UC) in nomogram and actual observation.

The AUC of CA125 was 0.60 (95% CI: 0.55–0.65, p < 0.01) and the AUC of HE4 was 0.52 (95% CI: 0.47–0.57, p = 0.43) for the diagnosis of UC. CA125 was significantly higher in patients with UC but not HE4. The sensitivity and specificity of CA125 with a cutoff of 50 U/ml was 32.5% and 96.3%. CA125, HE4, age, BMI, eGFR, smoking, NSAIDs, toxins, groundwater, and TCM were associated with UC in univariable logistic regression (Table 2) and were further analyzed using multivariable logistic regression. Age, eGFR, CA125, NSAIDs, toxins, smoking, and TCM were independently associated with UC. The OR was 1.09 (95% CI: 1.06–1.11, p < 0.01) for every one additional year, 1.03 (95% CI: 1.02–1.04, p < 0.01) per ml/min/1.73 m^2^ of eGFR, 3.03 (95% CI: 2.30–3.98) per log unit of CA125, 2.61 (95% CI: 1.37–4.97, p < 0.01) for smoking, 7.57 (95% CI: 3.81–15.03, p < 0.01) for NSAIDs, 3.33 (95% CI: 1.89–5.89, p < 0.01) for toxins, 8.25 (95% CI: 4.74–14.30, p < 0.01) for TCM. Age, eGFR, CA125, smoking, NSAIDs, toxins, and TCM were included in the nomogram Fig. 2. The sensitivity and specificity of the nomogram was 86.8% and 97.8%. The AUC of the nomogram was 0.91 and the goodness-of-fit index was 0.66. The nomogram was further internal validated using bootstrapping. As shown in Fig. 3, the X-axis is the predicted UC probability estimated by the nomogram and the Y-axis is the actual rates of UC. The solid line represents the ideal reference line that predicted UC corresponds to the actual outcome, and the dashed line represents the ideal estimation. The actual UC probability corresponded closely to the prediction of the nomogram. The calibration plot showed a good agreement between the prediction by nomogram and actual observation.

**Table 2:** Odds ratios (ORs) of possible risk factors for urothelial cancer.

Risk factors	Univariable			Multivariable		
	OR	95% CI		OR	95% CI	
Age	1.07	1.05	1.09	1.09	1.06	1.11
BMI	0.96	0.91	1.00	0.99	0.93	1.05
eGFR	1.00	1.00	1.01	1.03	1.02	1.04
CA125 (log)	2.46	2.08	2.92	3.03	2.30	3.98
HE4 (log)	1.39	1.00	1.95	1.44	0.70	2.97
Smoking	3.07	2.00	4.69	2.61	1.37	4.97
NSAIDs	8.53	5.31	13.68	7.57	3.81	15.03
Toxins	7.09	4.88	10.30	3.33	1.89	5.89
Groundwater	2.09	1.12	3.92	0.84	0.31	2.29
TCM	9.42	6.45	13.77	8.25	4.76	14.30

HE4: human epididymis protein 4, BMI: body mass index, eGFR: estimated glomerular filtration rate using CKD-EPI formula, NSAIDs: Nonsteroid Anti-inflammatory Drugs, Toxins: exposure to dye, paint, or organic solvent, TCM: use of traditional Chinese medicine.

## Discussion

This is the first study to develop a UC nomogram using commonly available tumor marker and clinical characteristics to identify UC in CKD patients who had a high risk of developing UC^33^. We investigated the individual accuracy of CA125 or HE4 to predict UC in CKD patients. CA125 can identify UC patients from CKD patients with a higher cutoff (50 U/ml) but HE4 cannot. The log transformation of CA125 and HE4 were used in logistic regression because CA125 and HE4 were not normally distributed (Table 2). To minimize the measurement bias, all measurements of CA125 and HE4 were performed in a central laboratory. Other confounders of UC may have limited effect of the diagnostic value of CA125 because the ORs of CA125 were similar in Model 2 (including eGFR) and Model 3 (including medical history). The low sensitivity (32.5%) of CA125 for the diagnosis of UC can be further improved by the combination of medical history including patients’ age, eGFR, and environmental carcinogen exposures. The medical history that is important for the diagnosis of UC includes a history of smoking, exposure to environmental toxins (dye, paint, and organic solvent), use of NSAIDs and use of TCM. The nomogram based on these risk factors revealed a good accuracy for the diagnosis of UC. The nomogram was further internal validated with bootstrapping technique. We are currently carrying out a prospective study to validate the usefulness of the nomogram in CKD patients.

The mean age of UC patients in this study is similar to the age of UC patients reported in previous studies^27,34–36^. All UC patients were, in fact, CKD patients by the definition of CKD because they had pathologic abnormalities in their urinary tracts; particularly if they received unilateral nephrectomy for upper urinary tract UC. However, this fact is often overlooked by urologists. UC patients were rarely referred to a nephrologist for regular follow up of renal function after surgery, as CKD patients did in clinical practice. Cancer risk in patients on dialysis had been extensively studied^37^ but little is known about the risk of dialysis in UC patients. After unilateral or bilateral nephrectomy, UC patients may reach advanced CKD stage and become dialysis dependent later on. This possibility reminds us to pay more attention to the follow up of renal function and care for CKD in UC patients after surgery.

Patients with a history of smoking are associated with UC and this fact is well supported by previous study^5,6^. An occupational exposure to dye, paint, or organic solvent is associated with UC and this is also well known from previous studies^30^. The most striking finding in this study is patients who ever used TCM have a much higher probability of developing UC (OR: 8.25). Traditional Chinese medicines may contain aristolochic acid (AA) and /or heavy metals. Aristolochic acid is known as an important risk factor for developing UC and CKD^11,38–40^ but it is difficult to identify a history of AA exposure directly by questionnaire alone. We can only use history of TCM prescription as a surrogate indicator, and 38.5% of UC patients vs. 6.2% of control patients (p < 0.01) reported a history of receiving TCM prescription. This percentage can be under-estimated because of the short memory span in elderly patients. The best evidence of exposure to AA containing herbs would be to identify AA-DNA adducts in the urine. Using mass spectrometry, we tried to identify AA-DNA adduct in the urine as a surrogate marker for exposure to AA containing TCM, but none of the urine samples of UC patients had detectable AA-DNA adducts. Although AA containing TCM had been banned for importation to Taiwan since 2003, nevertheless, it is known that once exposed to AA, the carcinogenic effect may last for 30 years or longer^11,41^.

There are some limitations to this study. First, we targeted CKD patients who are at high risk of UC^33^ and the risk of UC is increased in patients with lower renal function. However, the eGFR was positively associated with UC probability because most of the UC patients had a better renal function at the diagnosis of UC than those with CKD. The score of eGFR may be different when applying the nomogram in the general population. Second, a causal relationship between medical history and UC is difficult to prove because of the cross-sectional study design. Third, some patient selection bias cannot be completely avoided because control patients were recruited mainly from nephrology clinics while UC patients were recruited mostly during hospital admissions. Fourth, the number of patients with on-going UC in this study was relatively small. As this is an ongoing project, we will continue our recruiting program and further validate our UC biosignature in a larger cohort.

## Conclusions

CA125 is a useful tumor marker for the diagnosis of UC in CKD patients but not HE4. A nomogram based on serum CA125 level, age, renal function, smoking, history of exposure to environmental carcinogens, use of NSAIDs and use of traditional Chinese medicine reveals a high accuracy for predicting UC in CKD patients.
